# West Nile Virus in Horses as a Sentinel Host in One Health Surveillance: Epidemiological Insights and Future Perspectives

**DOI:** 10.3390/microorganisms14061263

**Published:** 2026-06-03

**Authors:** Paula Nistor, Livia Stanga, Vlad Iorgoni, Alexandru Gligor, Bogdan Florea, Vlad Cocioba, Ionica Iancu, Cosmin Horatiu Maris, Viorel Herman

**Affiliations:** 1 Department of Infectious Diseases and Preventive Medicine, Faculty of Veterinary Medicine, University of Life Sciences “King Mihai I” from Timişoara, 300645 Timişoara, Romania; paula.nistor@usvt.ro (P.N.); vlad.iorgoni@usvt.ro (V.I.); alexandru.gligor@usvt.ro (A.G.); ionica.iancu@usvt.ro (I.I.); viorel.herman@fmvt.ro (V.H.); 2 Doctoral School “Veterinary Medicine”, University of Life Sciences “King Mihai I” from Timişoara, Calea Aradului 119, 300645 Timişoara, Romania; bogdan-alexandru.florea.fmv@usvt.ro (B.F.); vlad-mihai.cocioba.fmv@usvt.ro (V.C.); 3 Discipline of Microbiology, Faculty of Medicine, “Victor Babes” University of Medicine and Pharmacy, Eftimie Murgu Square 2, 300041 Timişoara, Romania; 4 Department of Internal Medicine, University of Life Sciences “King Mihai I” from Timişoara, 300645 Timişoara, Romania; 5 Department of Animal Husbandry, University of Life Sciences “King Mihai I” from Timişoara, 300645 Timişoara, Romania; 6 Department of Forestry, Faculty of Engineering and Applied Technologies, University of Life Sciences “King Mihai I” from Timișoara, 300645 Timișoara, Romania; cosmin.maris@usvt.ro; 7 Academy of Romanian Scientists, Str. Ilfov, Nr. 3, Sector 5, 50044 Bucharest, Romania

**Keywords:** West Nile virus, horses, sentinel surveillance, One Health, arboviruses, epidemiology, seroprevalence, vector-borne diseases

## Abstract

West Nile virus (WNV) is a globally distributed mosquito-borne flavivirus with significant implications for both veterinary and public health. While horses are incidental dead-end hosts, their epidemiological role extends beyond clinical disease, as they can serve as effective sentinel hosts for detecting local viral circulation. Their frequent exposure to mosquito vectors, ability to mount measurable antibody responses, geographic stability, accessibility for monitoring, and the possibility of observation within managed owner–veterinarian systems make them particularly suitable for surveillance within a One Health framework. Evidence from Europe and the Americas demonstrates that equine seroprevalence and field surveillance can identify transmission hotspots, reveal silent circulation, and contribute to the understanding of spatial and temporal risk patterns. The review also addresses key limitations, including vaccination effects, flavivirus cross-reactivity, methodological heterogeneity, and challenges in interpreting serological data across different ecological contexts. Strengthening equine sentinel surveillance through standardized methodologies and integration with predictive and geospatial approaches may improve early warning capacity and support more effective control of WNV and other emerging arboviral diseases.

## 1. Introduction

West Nile virus (WNV) is an enveloped, positive-sense single-stranded RNA virus belonging to the *Flaviviridae* family, first identified in Uganda in 1937 [1]. Since its initial recognition, WNV has expanded from a geographically limited pathogen to one of the most widely distributed mosquito-borne viruses in the world, with endemic or recurrent transmission documented on all continents except Antarctica [2]. This expansion has been driven by a combination of ecological and anthropogenic processes, including climatic shifts, changes in vector habitat, movement of infected birds, increasing overlap between wildlife and domestic animal systems, and the intensification of environmental conditions favorable to mosquito proliferation [3,4].

The ecological maintenance of WNV depends on a bird–mosquito–bird transmission cycle in which ornithophilic mosquitoes, primarily species of the genus *Culex*, acquire infection from viremic birds and subsequently transmit the virus to new avian hosts [5]. In this cycle, birds act as the principal amplifying hosts, whereas horses and humans are regarded as incidental or dead-end hosts because they generally do not develop viremia sufficient to infect feeding mosquitoes [6]. Although horses do not contribute meaningfully to onward transmission, their infection reflects local mosquito activity and viral circulation [7].

In response to the increasing importance of WNV in both veterinary and public health, sentinel-based surveillance has become a key approach for early detection of viral circulation. A sentinel host is one whose exposure, infection, or disease occurrence provides actionable information about pathogen circulation in a given ecological setting [8]. Horses meet key criteria of effective sentinel hosts, including frequent exposure to mosquito vectors, geographic stability, and accessibility for monitoring [7,9].

The value of horses as sentinel hosts is particularly relevant within a One Health framework, where interactions between wildlife reservoirs, arthropod vectors, domestic animals, humans, and environmental conditions collectively shape the transmission dynamics of West Nile virus. In this interconnected system, horses occupy an intermediate and strategically informative position: although they do not contribute to viral amplification, their infection reflects local vector activity and ecological suitability for transmission. As such, equine populations provide a measurable and geographically meaningful indicator of viral circulation, linking environmental drivers and vector dynamics to potential human health risk. This integrative role enhances the capacity of surveillance systems to detect emerging transmission patterns and to support timely, evidence-based public health interventions [7,10].

While existing literature has primarily focused on the clinical presentation, diagnostic approaches, and pathophysiological mechanisms of WNV infection in horses, comparatively less attention has been given to their broader epidemiological significance within integrated surveillance systems. In particular, there is an increasing need for a concept-driven synthesis that explicitly addresses the role of horses as sentinel hosts, not merely as incidental hosts but as interpretable indicators of viral circulation within complex ecological networks. Such an approach is especially relevant in the context of emerging and re-emerging arboviral threats, where early detection and accurate risk assessment depend on the integration of data across animal, human, and environmental domains. Accordingly, this review examines WNV in horses through the lens of sentinel surveillance, with emphasis on their ecological and epidemiological relevance, the interpretative value of equine serological and clinical data, their integration within One Health surveillance frameworks, key methodological and conceptual limitations, and future directions for improving their application in predictive and coordinated disease monitoring systems.

### Literature Search Strategy and Review Methodology

This narrative review was conducted through a structured literature search performed in PubMed, Scopus, Web of Science, and Google Scholar databases. The search included combinations of the following keywords: “West Nile virus”, “horses”, “equine”, “sentinel surveillance”, “One Health”, “seroprevalence”, “arbovirus surveillance”, and “vector-borne diseases”.

Priority was given to peer-reviewed articles published in English that focused on equine epidemiology, serological surveillance, vector-host interactions, and integrated One Health surveillance systems. Both classical and recent studies were considered to capture the historical development and current epidemiological understanding of equine WNV surveillance.

Studies focused exclusively on clinical case descriptions without epidemiological relevance, non-peer-reviewed reports, and articles lacking sufficient methodological information were excluded. Additional references were identified through citation tracking of relevant articles.

## 2. Ecological Basis of West Nile Virus Transmission and the Position of Horses in the Transmission Network

**Figure 1:**
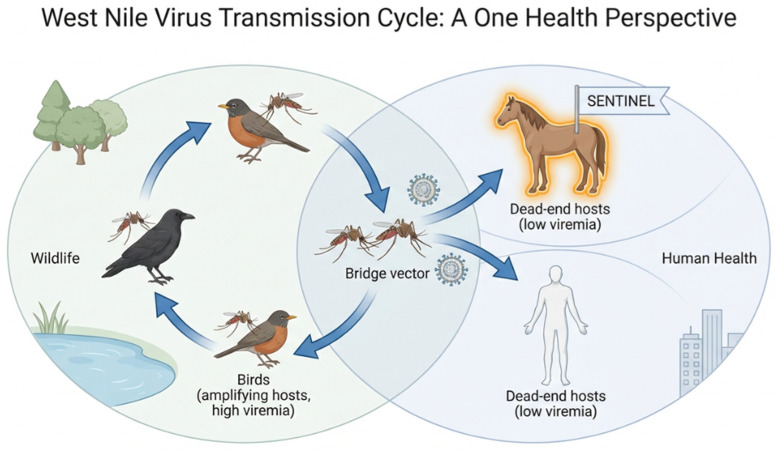
Ecological transmission cycle of West Nile virus and the sentinel position of horses within the One Health framework.

The suitability of horses as sentinel hosts cannot be understood without first considering the ecological organization of WNV transmission. At its core, WNV is maintained through a vector-borne cycle involving competent mosquitoes and susceptible avian hosts [11]. Mosquitoes acquire infection during blood feeding on viremic birds and can then transmit the virus to additional avian hosts, thereby maintaining enzootic circulation [12] (Figure 1). Spillover to horses and humans occurs when bridge vectors feed opportunistically on mammals, particularly in areas where mosquito density is high and virus amplification in birds has already occurred [13].

This transmission network is strongly shaped by environmental conditions, particularly temperature. Higher temperatures can accelerate mosquito development, increase feeding frequency, and shorten the extrinsic incubation period of West Nile virus within the vector, thereby increasing transmission potential under suitable ecological conditions. Rainfall and water availability determine larval habitat suitability, while land use, wetland density, vegetation structure, and urbanization influence both vector breeding sites and host contact patterns [14,15]. Environmental change, whether through climate warming, irrigation, altered drainage, urban sprawl, or habitat fragmentation, can increase the probability of contact between infected vectors and incidental hosts [16,17,18].

Birds occupy the central ecological role in WNV amplification and dispersal. Resident birds sustain local transmission cycles, whereas migratory birds may contribute to long-distance viral introduction [19]. Once the virus is introduced into a region that contains competent vectors and suitable ecological conditions, local transmission can become established. However, direct monitoring of avian hosts is often logistically difficult, incomplete, or biased toward visible mortality events. This is one reason why domestic species, especially horses, are so attractive in surveillance systems [20,21].

Horses sit at the interface between natural transmission ecology and managed animal health systems. Unlike migratory birds, they are usually geographically traceable. Unlike free-ranging wildlife, they can be sampled repeatedly and systematically. Unlike humans, they may reveal exposure patterns even in the absence of recognized outbreaks or intensive diagnostic activity. For these reasons, horses can provide a biologically meaningful and operationally feasible picture of local viral activity [22,23].

Another ecological advantage of horses is that their infection is generally interpreted as evidence of local mosquito-mediated exposure rather than long-range animal movement through reservoir pathways [23,24]. Although transport and management practices can complicate this interpretation in some contexts, equine seropositivity often carries stronger local ecological meaning than a sporadic human case with unknown exposure setting. Thus, the horse functions as an intermediate surveillance lens: biologically responsive to the ecosystem, but still sufficiently controlled and observable to generate high-value epidemiological information [25,26].

Nevertheless, interpretation of equine seropositivity as evidence of strictly local transmission may be complicated by horse movement associated with breeding, sport competitions, seasonal relocation, trade, or temporary housing. Accurate epidemiological interpretation therefore requires integration of animal movement history and management data whenever possible.

In practical terms, the horse does not replace avian or vector surveillance. Rather, it strengthens ecological inference by providing a measurable domestic host response to local transmission intensity. This makes equine surveillance particularly relevant in regions where avian monitoring is underdeveloped, where vector testing is intermittent, or where public health systems require supplementary indicators of transmission risk [4,27].

## 3. Why Horses Function as Sentinel Hosts for West Nile Virus Surveillance

**Figure 2:**
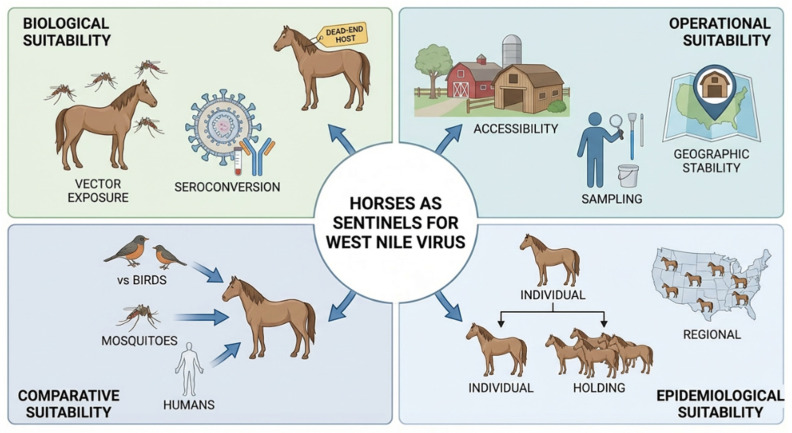
Biological, operational, epidemiological, and comparative factors supporting the role of horses as sentinel hosts for WNV surveillance.

The suitability of horses as sentinel hosts for WNV surveillance is the result of a convergence of biological, ecological, and operational characteristics that together provide a robust epidemiological signal [8,10]. This suitability can be understood through four complementary dimensions: biological, operational, epidemiological, and comparative suitability (Figure 2).

### 3.1. Biological and Epidemiological Suitability

The suitability of horses as sentinel species for arboviral surveillance is determined by the combination of biological responsiveness and epidemiological relevance. Horses are frequently exposed to mosquito vectors and develop detectable antibody responses following infection, making them reliable indicators of viral circulation. As dead-end hosts, they do not develop viremia sufficient to infect feeding mosquitoes, meaning that equine infection reflects local transmission without contributing to viral amplification [26,28,29,30,31,32].

From an epidemiological perspective, both serological and clinical findings in horses can provide information at multiple levels of inference. Detection of IgM antibodies may indicate recent exposure, whereas IgG seroprevalence reflects cumulative viral circulation over time. The spatial distribution of seropositive animals within holdings or regions can further support the identification of localized transmission patterns and regional risk assessment [33,34].

### 3.2. Operational Suitability

Operational suitability refers to the practicality of implementing equine-based surveillance under field conditions. Horses are generally owned, managed, and routinely monitored, which facilitates structured sampling and longitudinal follow-up. Their association with identifiable holdings enables accurate spatial attribution of epidemiological data, while veterinary oversight supports standardized clinical reporting and data collection. However, the effectiveness of this surveillance approach depends on stakeholder participation, including owner cooperation, accurate documentation of vaccination and movement history, and consistent involvement of veterinary services.

### 3.3. Comparative Suitability

Comparative suitability refers to the relative value of horses in relation to other components of WNV surveillance systems. Unlike birds, which serve as primary amplifying hosts but are difficult to monitor systematically, or mosquitoes, which provide direct evidence of viral presence but require technically demanding surveillance, horses offer a practical and interpretable host-based signal. Compared to human cases, which may be sporadic and associated with uncertain exposure locations, equine infections are often more geographically attributable. For these reasons, horses represent an intermediate and complementary surveillance component, bridging ecological processes and public health outcomes without replacing other surveillance modalities [35,36,37].

Nevertheless, equine surveillance cannot replace avian or entomological surveillance systems. Birds remain the principal amplifying hosts and may provide earlier evidence of viral amplification, while vector surveillance offers direct evidence of viral circulation in mosquito populations.

## 4. Epidemiological Insights from Equine Seroprevalence and Field Surveillance Studies

Equine seroprevalence studies represent one of the most informative tools for understanding the circulation of WNV across different ecological contexts. These studies provide insight not only into the presence of the virus but also into the intensity, duration, and spatial distribution of transmission [7,25,38].

### 4.1. Regional Variability and Global Comparisons

In Europe, seroprevalence studies have demonstrated relatively low to moderate exposure levels in many regions. For example, in Germany, WNV seroprevalence in horses has been reported at approximately 3.3% using virus neutralization testing. Similar values have been reported in Austria (~5.3%), Croatia (~3.4%), and Greece (~4.0%), suggesting focal or emerging transmission patterns [7,39,40,41,42].

In contrast, higher seroprevalence levels have been observed in regions with longer-standing endemic circulation, including parts of southeastern Europe where values may exceed 10–20% [26,43,44,45]. These differences reflect cumulative exposure over time, ecological suitability for vectors, and regional differences in surveillance intensity [43,44,45].

In South America, studies such as that conducted in Brazil have revealed substantially higher levels of arbovirus exposure, with more than 50% of horses showing antibodies to at least one arbovirus and 32.1% confirmed as WNV-positive by PRNT90 [46]. These findings highlight the complexity of arboviral ecology in tropical environments, where multiple viruses co-circulate and environmental conditions favor sustained transmission [25,26,38,39,40,41,42,43,44,45,46].

However, direct comparison between studies remains difficult because seroprevalence estimates are strongly influenced by differences in study design, sampling strategy, diagnostic assays, vaccination coverage, ecological conditions, and temporal context. Consequently, similar seroprevalence values may not necessarily indicate equivalent transmission intensity across regions.

### 4.2. Interpretation of Seroprevalence

**Figure 3:**
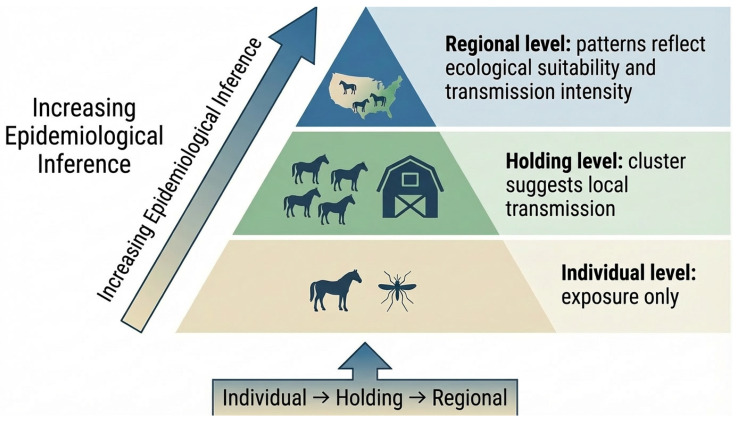
Multi-level interpretation of equine seroprevalence in WNV surveillance.

Seroprevalence must be interpreted within its epidemiological context, as at the individual level it indicates exposure without defining the timing or source of infection, at the holding level the presence of multiple seropositive animals suggests local transmission, and at the regional level overall seroprevalence patterns reflect ecological suitability and the intensity of viral circulation [25,26,47] (Figure 3).

Interpretation of equine seroprevalence should distinguish between endemic circulation, recent outbreak-associated transmission, and vaccine-induced seropositivity. High IgG seroprevalence in endemic regions may reflect long-term cumulative exposure rather than active transmission, whereas IgM detection is more indicative of recent viral circulation. In vaccinated populations, serological interpretation requires careful integration of vaccination history and confirmatory testing.

Importantly, seropositivity does not necessarily indicate recent circulation. IgG antibodies may persist for extended periods, meaning that seroprevalence reflects both past and current exposure. This limitation must be considered when using equine data for real-time risk assessment [25,26,48].

### 4.3. Determinants of Variability

Differences in seroprevalence across studies are influenced by multiple interacting factors, including ecological conditions such as temperature, water availability, and vector density, the presence and diversity of avian reservoir hosts, variations in study design (e.g., random versus convenience sampling), the vaccination status of horses, as well as the diagnostic methods used and the thresholds applied for defining seropositivity [13,25,48,49]. Age is one of the most consistent determinants, with older horses showing higher seropositivity due to cumulative exposure [47,50,51].

Furthermore, many published studies rely on convenience sampling rather than structured epidemiological designs, potentially introducing selection bias and limiting extrapolation to broader equine populations. Variability in laboratory confirmation protocols and serological thresholds further complicates inter-study comparability.

### 4.4. Detection of Silent Transmission

One of the most important contributions of equine surveillance is the detection of silent or cryptic transmission. In many regions, equine seroconversion has been documented in the absence of reported human cases, indicating that viral circulation may occur undetected by public health systems [26,32,44].

This feature makes horses particularly valuable in early-stage emergence or in areas with limited diagnostic infrastructure.

## 5. Clinical Relevance in Sentinel Surveillance: Why Clinical Disease Still Matters

Although a proportion of infected horses may develop neuroinvasive disease, this does not invalidate their role as sentinel hosts. In sentinel surveillance, the defining feature is not the absence of disease, but the epidemiological interpretability of exposure or infection. Horses remain valuable sentinels because both subclinical seroconversion and clinically apparent infection can indicate local viral circulation. Clinical disease represents the more visible end of the same transmission process and may serve as an important trigger for surveillance, particularly where active serological monitoring is limited [35,52].

Most WNV infections in horses are subclinical, but a minority of infected naïve horses develop neuroinvasive disease, often with signs such as ataxia, weakness, muscle fasciculations, cranial nerve deficits, and recumbency [53,54]. The fact that not all infections become clinically visible is actually advantageous from a sentinel perspective: equine populations can provide both silent serological evidence of circulation and more visible clinical alerts [55]. Together, these two forms of information create a richer surveillance signal than clinical reporting alone.

Clinical cases are especially important in regions where structured active surveillance is limited [35,47]. A horse presenting with acute neurological disease may serve as a practical epidemiological alarm that prompts testing, vector attention, and risk reassessment. In this sense, the horse functions as a sentinel not only because it seroconverts, but because its disease may be noticed, investigated, and reported [7,46,56].

The relationship between equine clinical disease and human risk is not always direct or linear [57,58]. A single equine case does not necessarily predict a human outbreak, and the absence of equine neurological disease does not exclude local circulation [30]. Nevertheless, the occurrence of confirmed equine cases adds important contextual value to surveillance and may justify enhanced attention to local mosquito activity and public health preparedness [51,59].

Therefore, while horses should not be valued as sentinels only through overt disease, the clinical manifestation of WNV remains an important part of their surveillance significance. Equine clinical cases bring visibility, urgency, and interpretive weight to surveillance systems that might otherwise rely only on background serology.

## 6. Diagnostic Interpretation in Sentinel Surveillance Contexts

Because this review is centered on sentinel surveillance rather than diagnostic methodology itself, the importance of diagnosis lies primarily in how it shapes epidemiological interpretation. In the context of horse-based surveillance, the goal is often not merely to determine whether an individual horse is infected, but to infer whether viral circulation is occurring in a given area and how confidently that inference can be made.

Serological testing forms the backbone of equine sentinel surveillance. Horses that seroconvert after exposure can reveal local transmission even in the absence of clinical signs [60,61]. However, the value of this information depends heavily on correct interpretation. IgM-based testing is particularly informative for recent infection, whereas IgG-based screening is more useful for cumulative exposure or seroprevalence estimation. Competitive ELISA assays are useful for large-scale screening, but their interpretation may be limited by flavivirus cross-reactivity, especially in regions where WNV, USUV, TBEV, or SLEV may overlap [62,63,64,65,66].

Considerable heterogeneity exists among serological assays used across studies, including differences in ELISA format, antigen composition, cutoff interpretation, and confirmatory neutralization criteria. Variability in PRNT thresholds and laboratory validation standards may substantially influence reported seroprevalence estimates and complicate direct epidemiological comparisons between regions (Table 1) [63,64,65,67,68,69].

The choice of diagnostic method depends on the epidemiological objective of surveillance, including detection of recent transmission, estimation of cumulative exposure, confirmation of WNV-specific antibodies, or ecological monitoring of vectors and reservoir hosts. Differences in assay sensitivity, specificity, and interpretive criteria must be considered when comparing surveillance data across studies and regions [55,66,70,71,72].

For sentinel surveillance, confirmatory neutralization testing is often essential. Plaque Reduction Neutralization Test (PRNT90) and related virus neutralization methods increase specificity and help distinguish WNV exposure from serological responses to related flaviviruses. This is particularly important when equine seropositivity is being used to guide public health or ecological inference, since false-positive or non-specific results may distort the perceived distribution of risk [66,67,68,69,70].

Another key issue is vaccination. In sentinel systems, vaccinated horses are often less useful as interpretable indicators of natural exposure unless surveillance is explicitly designed to account for immunization history. This is why several studies emphasize the value of using unvaccinated horses as sentinel populations [55,70,71,72].

Thus, the relevance of diagnostics in sentinel surveillance is not only technical but strategic. The surveillance value of the horse depends on whether test results can be interpreted as meaningful evidence of local viral circulation. Standardized testing algorithms and clear vaccination documentation are therefore as important as the assays themselves.

## 7. Integration of Horses into One Health Surveillance Systems

Effective WNV surveillance requires a multi-layered approach that integrates data from different biological and institutional domains. Within this framework, horses provide a critical intermediate layer linking ecological processes with public health outcomes.

### 7.1. Structure of an Integrated System

**Figure 4:**
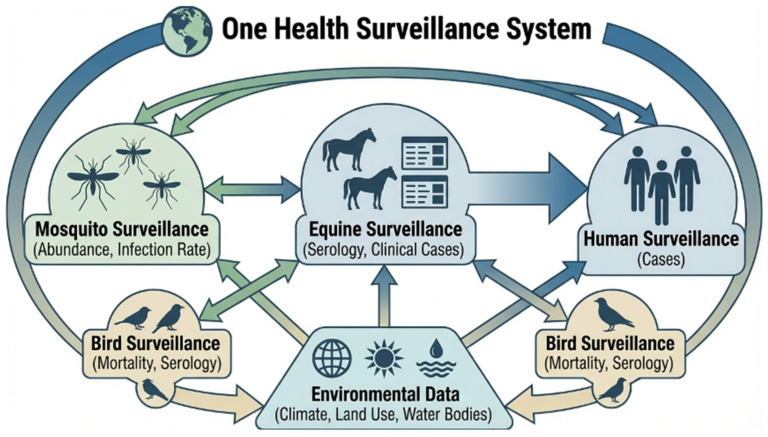
Integrated One Health surveillance system highlighting the role of horses as an intermediate epidemiological indicator.

A functional One Health surveillance system typically incorporates several complementary components, including entomological surveillance focused on mosquito abundance and infection rates, avian surveillance based on mortality monitoring and serology, equine surveillance through seroprevalence studies and clinical case detection, human surveillance involving clinical and laboratory-confirmed cases, and environmental data such as climate patterns, land use, and the distribution of water bodies [73,74,75]. Within this structure, horses contribute by translating ecological exposure into measurable biological responses, thereby connecting environmental risk with observable infection patterns in a domestic species (Figure 4).

### 7.2. Role of Equine Data

Equine data can be used to identify transmission hotspots, validate ecological risk models, detect early viral circulation, and trigger targeted interventions such as vector control measures or public health alerts [76,77]. The utility of horses as sentinel hosts is maximized when equine surveillance data are interpreted alongside entomological indicators such as mosquito abundance and infection rates, avian mortality and serology, human case surveillance, climatic and spatial risk mapping, and veterinary field observations. This integrated interpretation allows equine findings to be placed within a broader epidemiological context and enhances their predictive value [30,32].

### 7.3. Practical Implementation Challenges

Despite their epidemiological value, equine data are often not fully integrated into One Health surveillance systems. In practice, several barriers reduce their impact. These include fragmentation between veterinary and public health institutions, differences in reporting thresholds, lack of interoperable databases, inconsistent laboratory confirmation pathways, incomplete documentation of vaccination history, and delays in communication between field veterinarians, diagnostic laboratories, and public health authorities. In addition, the usefulness of equine surveillance depends on owner cooperation, local veterinary engagement, and the existence of clear protocols for sampling, reporting, and feedback. Without these structural elements, equine findings may remain isolated observations rather than actionable surveillance signals [9,78].

In several European surveillance systems, equine neurological case reporting has been integrated with mosquito testing and human surveillance databases to improve early warning capacity. However, interoperability between veterinary and public health information systems remains inconsistent across regions, limiting real-time epidemiological integration and coordinated response [9,32,78].

### 7.4. Examples of Integration

In several European countries, equine data have been incorporated into national surveillance programs, where the detection of equine cases can trigger enhanced mosquito surveillance or targeted public health alerts [4,79]. However, the degree of integration remains variable, and in many settings surveillance systems remain reactive, responding to detected cases rather than proactively identifying areas at risk. Strengthening the integration of equine data into predictive and coordinated One Health frameworks remains a key priority [80].

### 7.5. Role of Owners, Keepers, and Caretakers in Equine Sentinel Surveillance

The epidemiological value of equine sentinel surveillance depends partly on stakeholder participation, as owner consent and veterinary engagement directly influence sampling coverage, data completeness, and representativeness. Incomplete vaccination records, limited reporting of animal movements, and variable participation rates may introduce selection bias and affect the interpretation of seroprevalence estimates. Consequently, stakeholder-related factors should be considered as sources of epidemiological uncertainty when designing and interpreting equine surveillance studies. From this perspective, owner and veterinarian involvement is relevant not primarily as an operational component, but as a determinant of surveillance quality and data validity [80,81,82,83,84,85,86,87].

## 8. Limitations of Horses as Sentinel Hosts

**Figure 5:**
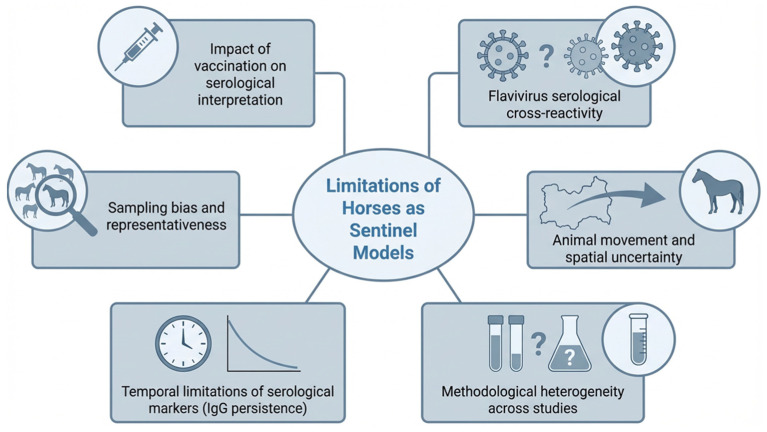
Major limitations affecting the use of horses as sentinel models in WNV surveillance.

While horses are valuable sentinel hosts, their use is associated with important limitations that must be critically considered in epidemiological interpretation and surveillance design [30,34,88,89,90] (Figure 5).

From a critical perspective, the usefulness of horses as sentinel models depends on a balance between biological interpretability and epidemiological relevance. While their status as dead-end hosts enhances the clarity of interpretation, it also limits their capacity to reflect transmission intensity in highly endemic settings, where repeated exposure may obscure temporal patterns. Moreover, in regions with widespread vaccination, serological data may no longer reliably indicate natural infection, further complicating interpretation. These factors highlight that the sentinel value of horses is conditional rather than absolute [25,44,55,90].

While numerous studies support the use of horses as sentinel models for WNV surveillance, the strength and consistency of this approach vary across ecological and epidemiological contexts. In particular, previous research has demonstrated that equine seroprevalence can provide an early indication of viral circulation in some settings, whereas in others its interpretative value is reduced by factors such as high baseline immunity, vaccination coverage, or complex transmission dynamics. Therefore, the role of horses as sentinel models should not be considered uniformly applicable, but rather context-dependent and influenced by local ecological and operational conditions [26,44,55].

Importantly, these limitations are not merely technical constraints but directly influence the epidemiological interpretation of equine surveillance data. For example, failure to account for vaccination status or cross-reactive antibody responses may lead to overestimation of transmission intensity, while uneven sampling may distort apparent spatial patterns. As a result, equine-based surveillance should be interpreted with caution and always integrated with complementary data sources, including entomological and human surveillance, to ensure accurate risk assessment [7,25,44,90].

### 8.1. Selection Bias and Representativeness

Many equine surveillance studies rely on convenience sampling rather than systematically designed sampling frameworks. As a result, the populations included may not accurately represent the broader equine population within a region. Differences in management systems, geographic distribution, and owner participation can introduce bias and affect the generalizability of findings [26,55].

### 8.2. Methodological Heterogeneity

Considerable variability exists across studies in terms of sampling strategies, diagnostic assays used, thresholds for defining seropositivity, and inclusion criteria. This heterogeneity limits comparability between studies and complicates attempts to synthesize data across regions or to draw robust conclusions about large-scale epidemiological patterns [86,87].

### 8.3. Serological Cross-Reactivity

Cross-reactivity between flaviviruses represents a major limitation in equine surveillance. In regions where WNV co-circulates with viruses such as Usutu virus, tick-borne encephalitis virus, or Saint Louis encephalitis virus, serological screening assays may detect antibodies that are not specific to WNV. Without confirmatory testing, this can lead to misclassification and overestimation of WNV exposure [64,88].

This limitation has important epidemiological implications, particularly in areas with complex flavivirus ecology. Cross-reactive antibody responses may obscure the true distribution and intensity of WNV circulation, leading to inaccurate identification of transmission hotspots and potentially misleading risk assessments. In addition, the extent of cross-reactivity may vary depending on the assay used, the antigenic similarity between circulating viruses, and the immune history of the individual animal, including prior exposure to other flaviviruses or vaccination [48,88].

From a surveillance perspective, failure to adequately address cross-reactivity can reduce the specificity and interpretability of equine sentinel data, especially when serological results are used to inform public health decisions or ecological modeling. For this reason, confirmatory testing using virus neutralization assays, such as PRNT, is essential in regions where multiple flaviviruses co-circulate. Moreover, integrating serological findings with ecological, entomological, and epidemiological data can help contextualize results and improve the accuracy of inference regarding local WNV activity [89,90].

### 8.4. Vaccination Effects

Vaccination further complicates serological interpretation. Vaccinated horses may develop antibody responses that are indistinguishable from those induced by natural infection in certain assays, particularly IgG-based tests. Unless vaccination history is accurately documented and incorporated into surveillance design, the sentinel value of equine serology may be reduced [91,92].

### 8.5. Mobility and Spatial Uncertainty

Although horses are generally more geographically stable than wildlife species, they may still be transported for competition, breeding, or trade. This mobility can obscure the true location of exposure and complicate spatial analyses that assume infection reflects local transmission [28,93].

### 8.6. Temporal Limitations

Seropositivity does not necessarily indicate recent transmission. The persistence of IgG antibodies means that positive results may reflect historical exposure rather than current viral circulation. Without complementary data, such as IgM detection or longitudinal sampling, it can be difficult to distinguish between past and ongoing transmission [42,94,95].

### 8.7. Economic and Logistical Constraints

The implementation of large-scale equine surveillance systems requires substantial resources, including financial investment, laboratory capacity, and veterinary infrastructure. These requirements are not uniformly met across regions, which may limit the feasibility and sustainability of equine-based surveillance programs, particularly in resource-limited settings [8,96].

### 8.8. Stakeholder Dependence and Participation Barriers

Unlike wildlife-based surveillance, equine sentinel systems depend heavily on owner consent, caretaker cooperation, and veterinary engagement. Limited awareness, concerns about the consequences of positive findings, incomplete vaccination records, reluctance toward repeated sampling, or logistical constraints at the holding level may reduce participation and introduce additional selection bias. These human factors can substantially affect the representativeness, continuity, and practical implementation of equine surveillance programs [73,97,98,99].

## 9. Future Perspectives

Within a One Health framework, the contribution of equine surveillance should be viewed as one component of a multi-layered system rather than a standalone indicator. Although horses provide valuable geographically anchored data, their interpretative value is maximized only when integrated with vector surveillance, wildlife monitoring, and human case reporting. This highlights the need for coordinated surveillance strategies that move beyond isolated data streams toward fully integrated epidemiological systems [7,25,44,99].

**Figure 6:**
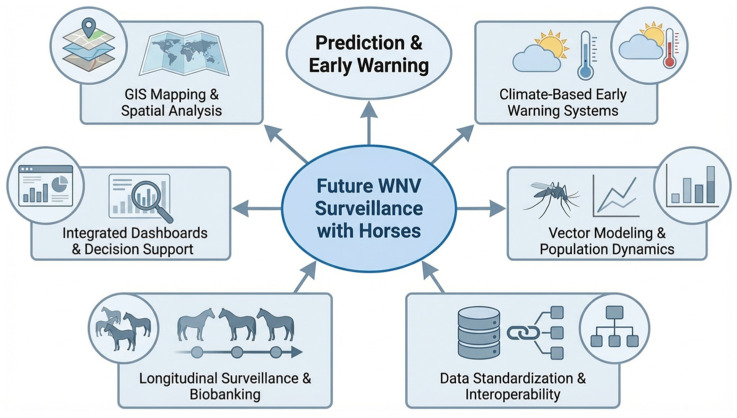
Emerging directions for integrating equine data into predictive and geospatial WNV surveillance systems.

The future value of horses in WNV surveillance will depend on making equine data more standardized, more comparable, and more integrated into predictive systems, as outlined in Figure 6.

Future research should focus not only on expanding equine surveillance but also on improving its analytical robustness and integration within broader surveillance frameworks. This includes standardization of serological methodologies, better differentiation between vaccination- and infection-derived antibodies, and the development of models that incorporate equine data alongside environmental and entomological variables. Such approaches would enhance the predictive value of equine surveillance and support its role in early warning systems.

One important direction is harmonization of surveillance protocols. Standard approaches to horse selection, vaccination assessment, sampling timing, screening assays, and confirmatory testing would improve comparability across studies and allow stronger epidemiological inference at regional and international levels [90].

Another promising direction is the increased use of geospatial epidemiology. Mapping equine seropositivity alongside climate variables, mosquito abundance, land-use data, water bodies, and bird migration routes could improve the spatial resolution of WNV risk prediction. Horses are especially suitable for this because their residence or holding location is often known with precision [92].

Longitudinal equine surveillance is also likely to be more informative than isolated cross-sectional studies. Repeated monitoring across transmission seasons can reveal temporal trends, identify emerging hotspots, and detect changes in endemicity. Such systems may be especially valuable in regions where WNV is expanding geographically or fluctuating in intensity [44].

Future research should also define the ecological conditions under which horses are most informative as sentinels. Their usefulness may vary according to vaccination prevalence, vector species composition, bird ecology, and local management systems. Moving from descriptive surveillance toward evidence-based sentinel optimization would strengthen the practical role of equine populations in WNV preparedness.

Finally, horses may also serve as models for broader arboviral sentinel surveillance. In regions where multiple flaviviruses circulate, equine surveillance could contribute to integrated monitoring systems capable of detecting not just WNV, but changes in overall arboviral pressure across domestic animal landscapes.

### 9.1. Limitations

This review is subject to certain limitations, including reliance on heterogeneous data sources and variability in study design across the literature. Differences in diagnostic methods, sampling strategies, and ecological contexts may affect the comparability of reported findings. Consequently, the conclusions presented here should be interpreted within the context of these constraints.

### 9.2. AI-Assisted Figure Preparation

All figures included in this manuscript were created by the authors using FigureLabs (https://www.figurelabs.ai/, accessed on 2 February 2026), an AI-assisted illustration tool, under an active paid subscription. The generated figures were subsequently reviewed, edited, and validated by the authors to ensure scientific accuracy, relevance, and consistency with the manuscript content. No previously published material was reproduced.

### 9.3. AI-Assisted Text Preparation Disclosure

During the preparation of this manuscript, the authors did not use artificial intelligence (AI) tools for the generation of scientific text, interpretation of data, or formulation of conclusions. AI-assisted tools were used exclusively for graphical illustration (FigureLabs), as described above. All written content, including conceptual development, literature synthesis, and critical interpretation, was produced, reviewed, and validated entirely by the authors. The authors take full responsibility for the integrity and originality of the manuscript in accordance with journal policies.

## 10. Conclusions

This review indicates that the value of horses in WNV surveillance extends beyond their role as incidental hosts and lies in their capacity to provide an epidemiologically interpretable signal of local viral circulation. The evidence reviewed across Europe and the Americas consistently shows that equine serological and clinical data can contribute to the identification of transmission hotspots, detection of silent circulation, and assessment of spatial risk patterns, particularly when integrated with entomological, environmental, and human surveillance data.

A central finding emerging from the literature is that the usefulness of horses as sentinel models is context-dependent rather than universal. Their surveillance value is influenced by ecological conditions, vaccination coverage, diagnostic methodologies, sampling design, and the degree of integration within One Health surveillance systems. Consequently, equine data should not be interpreted in isolation but as one component of a broader epidemiological framework that combines multiple complementary sources of evidence.

However, the surveillance value of horses is inseparable from the cooperation of owners, keepers, caretakers, and veterinarians, whose participation determines whether sentinel systems can be implemented consistently, interpreted correctly, and sustained over time. Expanding and refining equine surveillance through standardized methodology, integrated data sharing, and predictive epidemiological tools will enhance preparedness and improve the capacity of One Health systems to detect and respond to WNV circulation in a timely and meaningful way.

## Figures and Tables

**Table 1:** Diagnostic approaches used in West Nile virus surveillance and their epidemiological applications.

Diagnostic Category	Assay	Main Target	Sample Type	Main Epidemiological Application	Limitations
Molecular	RT-PCR	Viral RNA	Whole blood, CSF, mosquito pools, tissues	Detection of active infection	Short viremia period
Molecular	Real-time RT-PCR	Viral RNA	Serum, tissues, mosquito pools	Early detection and confirmation	Requires laboratory infrastructure
Serological	IgM ELISA	Recent antibody response	Serum	Detection of recent infection	Possible flavivirus cross-reactivity
Serological	IgG ELISA	Cumulative exposure	Serum	Seroprevalence studies	Cannot distinguish old infection
Confirmatory serology	Plaque Reduction Neutralization Test (PRNT)	Neutralizing antibodies	Serum	Confirmation of WNV-specific antibodies	Labor-intensive
Virus isolation	Cell culture/embryonated eggs	Infectious virus	Blood, tissues, mosquito pools	Research and strain characterization	Low sensitivity in horses

## Data Availability

No new data were created or analyzed in this study. Data sharing is not applicable to this article.

## Abbreviations

The following abbreviations are used in this manuscript:

WNV	West Nile virus
PRNT	Plaque Reduction Neutralization Test
PRNT90	90% Plaque Reduction Neutralization Test
ELISA	Enzyme-Linked Immunosorbent Assay
IgM	Immunoglobulin M
IgG	Immunoglobulin G
USUV	Usutu virus
GIS	Geographic Information System

## References

[1] Marino A., Vitale E., Maniaci A., La Via L., Moscatt V., Spampinato S., Senia P., Venanzi Rullo E., Restivo V., Cacopardo B. (2025). West Nile virus: Insights into microbiology, epidemiology, and clinical burden. Acta Microbiol. Hell..

[2] Habarugira G., Suen W.W., Hobson-Peters J., Hall R.A., Bielefeldt-Ohmann H. (2020). West Nile virus: An update on pathobiology, epidemiology, diagnostics, control and One Health implications. Pathogens.

[3] Ferraguti M., Martínez-de la Puente J., Figuerola J. (2021). Ecological effects on the dynamics of West Nile virus and avian *Plasmodium*: The importance of mosquito communities and landscape. Viruses.

[4] Nistor P., Stanga L., Iorgoni V., Cojocaru R.G., Gligor A., Ciresan A., Florea B., Cocioba V., Iancu I., Iorgoni H. (2026). West Nile virus in Europe: Epidemiology, vector ecology, environmental drivers, and the role of equine sentinel surveillance in a One Health framework. Pathogens.

[5] Wang Y., Calzolari M., Calvi G., Cox V.M., Angelini P., Dottori M., Wint W., Jahn S., Marini G., Dorigatti I. (2026). Association of avian biodiversity and West Nile virus circulation in *Culex* mosquitoes in Emilia-Romagna, Italy. PLoS Negl. Trop. Dis..

[6] Taieb L., Ludwig A., Ogden N.H., Lindsay R.L., Iranpour M., Gagnon C.A., Bicout D.J. (2020). Bird species involved in West Nile virus epidemiological cycle in southern Québec. Int. J. Environ. Res. Public Health.

[7] Gothe L.M.R., Ganzenberg S., Ziegler U., Obiegala A., Lohmann K.L., Sieg M., Vahlenkamp T.W., Groschup M.H., Hörügel U., Pfeffer M. (2023). Horses as sentinels for the circulation of flaviviruses in Eastern-Central Germany. Viruses.

[8] Najafi S., Jojani M., Najafi K., Costanzo V., Vicidomini C., Roviello G.N. (2026). West Nile virus: Epidemiology, surveillance, and prophylaxis with a comparative insight from Italy and Iran. Vaccines.

[9] Nistor P., Stânga L., Chirilă A., Iorgoni V., Cocioba V., Cojocaru R.G., Gligor A., Cireșan A., Florea B., Iorgoni H. (2026). Gaps between awareness and prevention of West Nile virus among horse owners in an endemic country: A cross-sectional study from Romania. Vet. Sci..

[10] Alibekova D.A., Barakbayev K.B., Omarova Z.D., Rystaeva R.A., Sultankulova K.T., Burashev Y.D., Argimbayeva T.U., Tulendibayev A.B., Aubakir N.A., Yermekbay T.T. (2025). Serological investigations on West Nile virus in horses in Kazakhstan. Microorganisms.

[11] Mencattelli G., Silverj A., Iapaolo F., Ippoliti C., Teodori L., Di Gennaro A., Curini V., Candeloro L., Conte A., Polci A. (2022). Epidemiological and evolutionary analysis of West Nile virus lineage 2 in Italy. Viruses.

[12] Pauli G., Bauerfeind U., Blümel J., Burger R., Drosten C., Gröner A., Gürtler L., Heiden M., Hildebrandt M., Jansen B. (2013). West Nile virus. Transfus. Med. Hemother..

[13] Vargas Campos C.A., García-Pérez S., Figuerola J., Martínez-de la Puente J., Polo I., Rodríguez-de-Fonseca B., Fernández-Álvarez S., Galván Fraile V., Martín-Rey M., Lacasaña M. (2025). Comprehensive analysis of West Nile virus transmission: Environmental, ecological, and individual factors. An umbrella review. One Health.

[14] Kilpatrick A.M., Meola M.A., Moudy R.M., Kramer L.D. (2008). Temperature, viral genetics, and the transmission of West Nile virus by Culex pipiens mosquitoes. PLoS Pathog..

[15] Reisen W.K., Fang Y., Martinez V.M. (2006). Effects of temperature on the transmission of West Nile virus by Culex tarsalis (Diptera: Culicidae). J. Med. Entomol..

[16] Pascoe L., Clemen T., Bradshaw K., Nyambo D. (2022). Review of importance of weather and environmental variables in agent-based arbovirus models. Int. J. Environ. Res. Public Health.

[17] Montgomery M.J., Harwood J.F., Yougang A.P., Wilson-Bahun T.A., Tedjou A.N., Keumeni C.R., Wondji C.S., Kamgang B., Kilpatrick A.M. (2025). The effects of urbanization, temperature, and rainfall on *Aedes aegypti* and *Aedes albopictus* mosquito abundance across a broad latitudinal gradient in Central Africa. Parasites Vectors.

[18] Nayak P.P., Pai B.J., Govindan S., Babu N.N. (2025). Influence of climatic and land use factors on post-monsoon distribution of *Aedes* mosquito vectors in Udupi taluk. Sci. Rep..

[19] Kilpatrick A.M., LaDeau S.L., Marra P.P. (2007). Ecology of West Nile virus transmission and its impact on birds in the Western Hemisphere. Auk.

[20] Geraldes M.A., Giovanetti M., Cunha M.V., Lourenço J. (2026). Land and climate suitability for West Nile virus in Atlantic archipelagos guided by historical data from Europe. Commun. Biol..

[21] Sofia M., Giannakopoulos A., Giantsis I.A., Touloudi A., Birtsas P., Papageorgiou K., Athanasakopoulou Z., Chatzopoulos D.C., Vrioni G., Galamatis D. (2022). West Nile virus occurrence and ecological niche modeling in wild bird species and mosquito vectors: An active surveillance program in the Peloponnese region of Greece. Microorganisms.

[22] Lönker N.S., Fechner K., Wahed A.A.E. (2020). Horses as a crucial part of One Health. Vet. Sci..

[23] Anderson B.D., Barnes A.N., Umar S., Guo X., Thongthum T., Gray G.C., Sing A. (2023). Reverse zoonotic transmission (zooanthroponosis): An increasing threat to animal health. Zoonoses: Infections Affecting Humans and Animals.

[24] Humphreys J.M., Pelzel-McCluskey A.M., Cohnstaedt L.W., McGregor B.L., Hanley K.A., Hudson A.R., Young K.I., Peck D., Rodriguez L.L., Peters D.P.C. (2021). Integrating spatiotemporal epidemiology, eco-phylogenetics, and distributional ecology to assess West Nile disease risk in horses. Viruses.

[25] Ganzenberg S., Sieg M., Ziegler U., Pfeffer M., Vahlenkamp T.W., Hörügel U., Groschup M.H., Lohmann K.L. (2022). Seroprevalence and risk factors for equine West Nile virus infections in Eastern Germany, 2020. Viruses.

[26] Nistor P., Stanga L., Chirila A., Iorgoni V., Gligor A., Ciresan A., Popa I., Florea B., Imre M., Cocioba V. (2025). Seroprevalence and passive clinical surveillance of West Nile virus in horses from ecological high-risk areas in Western Romania: Exploratory findings from a cross-sectional study. Microorganisms.

[27] Faverjon C. (2017). Risk Based Surveillance for Vector-Borne Diseases in Horses: Combining Multiple Sources of Evidence to Improve Decision Making. Ph.D. Thesis.

[28] Nistor P., Stanga L., Chirila A., Iorgoni V., Gligor A., Ciresan A., Florea B., Bota C., Cocioba V., Popa I. (2025). Mosquito exposure risks in equine facilities: An environmental–managerial assessment in Western Romania. Microorganisms.

[29] Boukraa S., de La Grandiere M.A., Bawin T., Raharimalala F.N., Zimmer J.Y., Haubruge E., Thiry E., Francis F. (2016). Diversity and ecology survey of mosquitoes potential vectors in Belgian equestrian farms: A threat prevention of mosquito-borne equine arboviruses. Prev. Vet. Med..

[30] Schwarz E.R., Long M.T. (2023). Comparison of West Nile virus disease in humans and horses: Exploiting similarities for enhancing syndromic surveillance. Viruses.

[31] Carrasco L., Utrilla M.J., Fuentes-Romero B., Fernandez-Novo A., Martin-Maldonado B. (2024). West Nile virus: An update focusing on Southern Europe. Microorganisms.

[32] García-Carrasco J.M., Muñoz A.R., Olivero J., Segura M., García-Bocanegra I., Real R. (2023). West Nile virus in the Iberian Peninsula: Using equine cases to identify high-risk areas for humans. Euro Surveill..

[33] Bergmann F., Trachsel D.S., Stoeckle S.D., Bernis Sierra J., Lübke S., Groschup M.H., Gehlen H., Ziegler U. (2022). Seroepidemiological survey of West Nile virus infections in horses from Berlin/Brandenburg and North Rhine-Westphalia, Germany. Viruses.

[34] Gonzálvez M., Franco J.J., Barbero-Moyano J., Caballero-Gómez J., Ruano M.J., Martínez R., Cano-Terriza D., García-Bocanegra I. (2023). Monitoring the epidemic of West Nile virus in equids in Spain, 2020–2021. Prev. Vet. Med..

[35] Leblond A., Lecollinet S. (2017). Clinical screening of horses and early warning for West Nile virus. Equine Vet. Educ..

[36] Bruno L., Nappo M.A., Frontoso R., Perrotta M.G., Di Lecce R., Guarnieri C., Ferrari L., Corradi A. (2025). West Nile virus (WNV): One-Health and Eco-Health global risks. Vet. Sci..

[37] Taheri S., González M., Ruiz-Lopez M.J., Soriguer R., Figuerola J. (2025). Patterns of West Nile virus vector co-occurrence and spatial overlap with human cases across Europe. One Health.

[38] Chevalier N., Migné C.V., Mariteragi-Helle T., Dumarest M., De Mas M., Chevrier M., Queré E., Marcillaud-Pitel C., Lupo C., Bigeard C. (2025). Seroprevalence of West Nile, Usutu and tick-borne encephalitis viruses in equids from south-western France in 2023. Vet. Res..

[39] De Heus P., Kolodziejek J., Hubálek Z., Dimmel K., Racher V., Nowotny N., Cavalleri J. (2021). West Nile virus and tick-borne encephalitis virus are endemic in equids in Eastern Austria. Viruses.

[40] Barbić L., Listeš E., Katić S., Stevanović V., Madić J., Starešina V., Labrović A., Di Gennaro A., Savini G. (2012). Spreading of West Nile virus infection in Croatia. Vet. Microbiol..

[41] Vilibić-Čavlek T., Savić V., Klobučar A., Ferenc T., Ilić M., Bogdanić M., Tabain I., Stevanović V., Santini M., Posavec M. (2021). Emerging trends in the West Nile virus epidemiology in Croatia in the ‘One Health’ context, 2011–2020. Trop. Med. Infect. Dis..

[42] Bouzalas I., Diakakis N., Chaintoutis S., Brellou G., Papanastassopoulou M., Danis K., Vlemmas I., Seuberlich T., Dovas C. (2016). Emergence of equine West Nile encephalitis in Central Macedonia, Greece, 2010. Transbound. Emerg. Dis..

[43] Metz M., Olufemi O., Daly J., Barba M. (2020). Systematic review and meta-analysis of seroprevalence studies of West Nile virus in equids in Europe between 2001 and 2018. Transbound. Emerg. Dis..

[44] Abbas I., Ahmed F., Muqaddas H., Alberti A., Varcasia A., Sedda L. (2025). Epidemiology and surveillance of West Nile virus in the Mediterranean Basin during 2010–2023: A systematic review. Curr. Res. Parasitol. Vector Borne Dis..

[45] Oslobanu L.E.L., Păslaru A., Savuța G. (2015). West Nile virus seroprevalence in horses from Romania: First step in the infection risk assessment. Bull. UASVM Vet. Med..

[46] Guimarães M.C.N., Freitas M.N.O., Sousa A.W., Cunha M.A.C.R., Almada G.L., Romano A.P.M., Santos M.G.D.P., Rodrigues G.A.P., Martins L.C., Chiang J.O. (2022). Serological evidence of arboviruses in horses during West Nile fever monitoring surveillance in Southeastern Brazil. Front. Trop. Dis..

[47] Selim A., Megahed A., Kandeel S., Alouffi A., Almutairi M.M. (2021). West Nile virus seroprevalence and associated risk factors among horses in Egypt. Sci. Rep..

[48] Beck C., Lowenski S., Durand B., Bahuon C., Zientara S., Lecollinet S. (2017). Improved reliability of serological tools for the diagnosis of West Nile fever in horses within Europe. PLoS Negl. Trop. Dis..

[49] Al-Rammahi H.M., Mohsen R.K., Othman R.M. (2025). First detection of West Nile virus seropositivity in horses in southern Iraq. Open Vet. J..

[50] Sule W.F., Oluwayelu D.O., Adedokun R.A., Rufai N., McCracken F., Mansfield K.L., Johnson N. (2015). High seroprevalence of West Nile virus antibodies observed in horses from southwestern Nigeria. Vector Borne Zoonotic Dis..

[51] Magallanes S., Llorente F., Ruiz-López M.J., Martínez-de la Puente J., Soriguer R., Calderon J., Jiménez-Clavero M.A., Aguilera-Sepúlveda P. (2023). Long-term serological surveillance for West Nile and Usutu virus in horses in south-West Spain. One Health.

[52] Cantile C., Di Guardo G., Eleni C., Arispici M. (2000). Clinical and neuropathological features of West Nile virus equine encephalomyelitis in Italy. Equine Vet. J..

[53] Fehér O.E., Fehérvári P., Tolnai C.H., Forgách P., Malik P., Jerzsele Á., Wagenhoffer Z., Szenci O., Korbacska-Kutasi O. (2022). Epidemiology and clinical manifestation of West Nile virus infections of equines in Hungary, 2007–2020. Viruses.

[54] Paré J., Moore A. (2018). West Nile virus in horses—What do you need to know to diagnose the disease?. Can. Vet. J..

[55] Naveed A., Eertink L.G., Wang D., Li F. (2024). Lessons learned from West Nile virus infection: Vaccinations in equines and their implications for One Health approaches. Viruses.

[56] Cleton N.B., van Maanen K., Bergervoet S.A., Bon N., Beck C., Godeke G.J., Lecollinet S., Bowen R., Lelli D., Nowotny N. (2017). A serological protein microarray for detection of multiple cross-reactive flavivirus infections in horses for veterinary and public health surveillance. Transbound. Emerg. Dis..

[57] Ward M.P., Scheurmann J.A. (2008). The relationship between equine and human West Nile virus disease occurrence. Vet. Microbiol..

[58] Laperriere V., Brugger K., Rubel F. (2011). Simulation of the seasonal cycles of bird, equine and human West Nile virus cases. Prev. Vet. Med..

[59] Riccardo F., Bolici F., Fafangel M., Jovanovic V., Socan M., Klepac P., Plavsa D., Vasic M., Bella A., Diana G. (2020). West Nile virus in Europe: After action reviews of preparedness and response to the 2018 transmission season in Italy, Slovenia, Serbia and Greece. Glob. Health.

[60] Folly A.J., Waller E.S.L., McCracken F., McElhinney L.M., Roberts H., Johnson N. (2020). Equine seroprevalence of West Nile virus antibodies in the UK in 2019. Parasites Vectors.

[61] El Garch H., Minke J.M., Rehder J., Richard S., Edlund Toulemonde C., Dinic S., Andreoni C., Audonnet J.C., Nordgren R., Juillard V. (2008). A West Nile virus recombinant canarypox virus vaccine elicits WNV-specific neutralizing antibodies and cell-mediated immune responses in the horse. Vet. Immunol. Immunopathol..

[62] Carson P.J., Prince H.E., Biggerstaff B.J., Lanciotti R., Tobler L.H., Busch M. (2014). Characteristics of antibody responses in West Nile virus-seropositive blood donors. J. Clin. Microbiol..

[63] Ledermann J.P., Lorono-Pino M.A., Ellis C., Saxton-Shaw K.D., Blitvich B.J., Beaty B.J., Bowen R.A., Powers A.M. (2011). Evaluation of widely used diagnostic tests to detect West Nile virus infections in horses previously infected with St. Louis encephalitis virus or dengue virus type 2. Clin. Vaccine Immunol..

[64] Beck C., Jimenez-Clavero M.A., Leblond A., Durand B., Nowotny N., Leparc-Goffart I., Zientara S., Jourdain E., Lecollinet S. (2013). Flaviviruses in Europe: Complex circulation patterns and their consequences for the diagnosis and control of West Nile disease. Int. J. Environ. Res. Public Health.

[65] Vilibic-Cavlek T., Ferenc T., Vujica Ferenc M., Bogdanic M., Potocnik-Hunjadi T., Sabadi D., Savic V., Barbic L., Stevanovic V., Monaco F. (2022). Cross-reactive antibodies in tick-borne encephalitis: Case report and literature review. Antibodies.

[66] Ziegler U., Angenvoort J., Klaus C., Nagel-Kohl U., Sauerwald C., Thalheim S., Horner S., Braun B., Kenklies S., Tyczka J. (2013). Use of competition ELISA for monitoring of West Nile virus infections in horses in Germany. Int. J. Environ. Res. Public Health.

[67] Magnarelli L.A., Bushmich S.L., Anderson J.F., Ledizet M., Koski R.A. (2008). Serum antibodies to West Nile virus in naturally exposed and vaccinated horses. J. Med. Microbiol..

[68] Cavalleri J.-M., Korbacska-Kutasi O., Leblond A., Paillot R., Pusterla N., Steinmann E., Tomlinson J. (2022). European College of Equine Internal Medicine consensus statement on equine flaviviridae infections in Europe. J. Vet. Intern. Med..

[69] Ben Mostafa K., Savini G., Dayhum A., Eldaghayes I. (2023). First detection of West Nile virus antibodies in animals in Libya. Int. J. Infect. Dis..

[70] Cendejas P.M., Goodman A.G. (2024). Vaccination and control methods of West Nile virus infection in equids and humans. Vaccines.

[71] Bahuon C., Marcillaud-Pitel C., Bournez L., Leblond A., Beck C., Hars J., Leparc-Goffart I., L’Ambert G., Paty M.C., Cavalerie L. (2016). West Nile virus epizootics in the Camargue (France) in 2015 and reinforcement of surveillance and control networks. Rev. Sci. Tech..

[72] Pearce M., Venter M., Schouwstra T., van Eeden C., van Vuren P.J., Liu B., du Plessis A. (2013). Serum neutralising antibody response of seronegative horses against lineage 1 and lineage 2 West Nile virus following vaccination with an inactivated lineage 1 West Nile virus vaccine. J. S. Afr. Vet. Assoc..

[73] Branda F., Ahmed M.M., Yon D.K., Ceccarelli G., Ciccozzi M., Scarpa F. (2025). Retrospective analysis and cross-validated forecasting of West Nile virus transmission in Italy: Insights from climate and surveillance data. Trop. Med. Infect. Dis..

[74] Todoric D., Vrbova L., Mitri M.E., Gasmi S., Stewart A., Connors S., Zheng H., Bourgeois A.C., Drebot M., Paré J. (2022). An overview of the National West Nile Virus Surveillance System in Canada: A One Health approach. Can. Commun. Dis. Rep..

[75] https://hal.science/hal-03712527v1/file/601300.pdf.

[76] Epp T.Y., Waldner C., Berke O. (2011). Predictive risk mapping of West Nile virus (WNV) infection in Saskatchewan horses. Can. J. Vet. Res..

[77] Geraldes M.A., Cunha M.V., Godinho C., de Lima R.F., Giovanetti M., Lourenço J. (2024). The historical ecological background of West Nile virus in Portugal indicates One Health opportunities. Sci. Total Environ..

[78] George J., Häsler B., Mremi I., Sindato C., Mboera L., Rweyemamu M., Mlangwa J. (2020). A systematic review on integration mechanisms in human and animal health surveillance systems with a view to addressing global health security threats. One Health Outlook.

[79] Patzina-Mehling C., Kopp A., Rauhöft L., Șuleșco T., Jones T.C., Drosten C., Sauer F.G., Lühken R., Junglen S. (2025). Genomic surveillance indicates high site-specific heterogeneity of West Nile virus in mosquitoes in rural regions of Germany across seasons. One Health.

[80] Özcelik R., Remy-Wohlfender F., Küker S., Visschers V., Hadorn D., Dürr S. (2021). Potential and challenges of community-based surveillance in animal health: A pilot study among equine owners in Switzerland. Front. Vet. Sci..

[81] Ince O., Paksoy Y., Sait A. (2024). Risk assessment about effectiveness of biosecurity implementations on horse properties in Turkey. J. Hell. Vet. Med. Soc..

[82] White N., Flynn K. (2024). Survey of state animal health officials reveals lack of personnel and resources to manage equine infectious disease outbreaks. J. Am. Vet. Med. Assoc..

[83] Costa E., Bayeux J., Silva A., De Queiroz G., Santos B., Rocha M., Rehfeld I., De Souza Franklin L., Valle L., Guedes M. (2020). Epidemiological surveillance of West Nile virus in the world and Brazil. Braz. J. Vet. Res. Anim. Sci..

[84] Struchen R., Hadorn D., Wohlfender F., Balmer S., Süptitz S., Zinsstag J., Vial F. (2016). Experiences with a voluntary surveillance system for early detection of equine diseases in Switzerland. Epidemiol. Infect..

[85] Grajeda L., McCracken J., Berger-González M., Lopez M., Alvarez D., Méndez S., Pérez O., Cordón-Rosales C., Zinsstag J. (2021). Sensitivity and representativeness of one-health surveillance for diseases of zoonotic potential at health facilities relative to household visits in rural Guatemala. One Health.

[86] Nistor P., Stanga L., Iorgoni V., Gligor A., Ciresan A., Iorgoni H., Florea B., Cocioba V., Iancu I., Maris C.H. (2026). Diagnosis and Surveillance of West Nile Virus Infection in Horses: Current Methods, Challenges, and Future Directions. Vet. Sci..

[87] Rusenova N., Rusenov A., Chervenkov M., Sirakov I. (2024). Seroprevalence of West Nile virus among equids in Bulgaria in 2022 and assessment of some risk factors. Vet. Sci..

[88] Chan K.R., Ismail A.A., Thergarajan G., Raju C.S., Yam H.C., Rishya M., Sekaran S.D. (2022). Serological cross-reactivity among common flaviviruses. Front. Cell. Infect. Microbiol..

[89] Ouni A., Aounallah H., Rebai W., Llorente F., Chendoul W., Hammami W., Rhim A., Jiménez-Clavero M., Pérez-Ramírez E., Bouattour A. (2025). The role of ruminants as sentinel animals in the circulation of the West Nile virus in Tunisia. Pathogens.

[90] Olufemi O., Barba M., Daly J. (2021). A scoping review of West Nile virus seroprevalence studies among African equids. Pathogens.

[91] Monaco F., Purpari G., Gennaro A., Mira F., Di Marco P., Guercio A., Savini G. (2019). Immunological response in horses following West Nile virus vaccination with inactivated or recombinant vaccine. Vet. Ital..

[92] Khatibzadeh S.M., Gold C.B., Keggan A.E., Perkins G.A., Glaser A.L., Dubovi E.J., Wagner B. (2015). West Nile virus-specific immunoglobulin isotype responses in vaccinated and infected horses. Am. J. Vet. Res..

[93] Aharonson-Raz K., Lichter-Peled A., Tal S., Gelman B., Cohen D., Klement E., Steinman A. (2014). Spatial and temporal distribution of West Nile virus in horses in Israel (1997–2013)—From endemic to epidemics. PLoS ONE.

[94] Williams R.A.J., Criollo Valencia H.A., López Márquez I., González González F., Llorente F., Jiménez-Clavero M.Á., Busquets N., Mateo Barrientos M., Ortiz-Díez G., Ayllón Santiago T. (2024). West Nile virus seroprevalence in wild birds and equines in Madrid Province, Spain. Vet. Sci..

[95] Faverjon C., Vial F., Andersson M.G., Lecollinet S., Leblond A. (2017). Early detection of West Nile virus in France: Quantitative assessment of syndromic surveillance system using nervous signs in horses. Epidemiol. Infect..

[96] Humblet M.-F., Vandeputte S., Fecher-Bourgeois F., Léonard P., Gosset C., Balenghien T., Durand B., Saegerman C. (2016). Estimating the economic impact of a possible equine and human epidemic of West Nile virus infection in Belgium. Euro Surveill..

[97] Farchati H., Merlin A., Saussac M., Dornier X., Dhollande M., Garon D., Tapprest J., Sala C. (2020). Is the French SIRE equine information system a good basis for surveillance and epidemiological research? Quality assessment using two surveys. Res. Vet. Sci..

[98] Bertram F.-M., Thompson P.N., Venter M. (2021). Epidemiology and clinical presentation of West Nile virus infection in horses in South Africa, 2016–2017. Pathogens.

[99] Chevalier V., Tran A., Durand B. (2014). Predictive modeling of West Nile virus transmission risk in the Mediterranean Basin: How far from landing?. Int. J. Environ. Res. Public Health.

